# Indigenous Ammonia-Oxidizing Archaea in Oxic Subseafloor Oceanic Crust

**DOI:** 10.1128/mSystems.00758-19

**Published:** 2020-03-10

**Authors:** Rui Zhao, Håkon Dahle, Gustavo A. Ramírez, Steffen L. Jørgensen

**Affiliations:** a Department of Biology, K.G. Jebsen Centre for Deep Sea Research, University of Bergen, Bergen, Norway; b Department of Marine Sciences, University of North Carolina at Chapel Hill, Chapel Hill, North Carolina, USA; c College of Veterinary Medicine, Western University of Health Sciences, Pomona, California, USA; d Department of Earth Science, K.G. Jebsen Centre for Deep Sea Research, University of Bergen, Bergen, Norway; Woods Hole Oceanographic Institution

**Keywords:** nitrification, Thaumarchaeota, deep biosphere, oceanic crust

## Abstract

Oceanic ridge flank systems represent one of the largest and least-explored microbial habitats on Earth. Fundamental ecological questions regarding community activity, recruitment, and succession in this environment remain unanswered. Here, we investigated ammonia-oxidizing archaea (AOA) in the sediment-buried basalts on the oxic and young ridge flank at North Pond, a sediment-filled pond on the western flank of the Mid-Atlantic Ridge, and compared them with those in the overlying sediments and bottom seawater. Nitrification in the North Pond basement is thermodynamically favorable and is supported by a reaction-transport model simulating the dynamics of nitrate in the crustal fluids. Nitrification rate is estimated to account for 6% to 7% of oxygen consumption, which is similar to the ratios found in marine oxic sediments, suggesting that aerobic mineralization of organic matter is the major ammonium source for crustal nitrifiers. Using the archaeal 16S rRNA and *amoA* genes as phylogenetic markers, we show that AOA, composed solely of Nitrosopumilaceae, are the major archaeal dwellers at North Pond. Phylogenetic analysis reveals that the crustal AOA communities are distinct from those in the bottom seawater and the upper oxic sediments but are similar to those in the basal part of the overlying sediment column, suggesting either similar environmental selection or the dispersal of microbes across the sediment-basement interface. Additionally, quantitative abundance data suggest enrichment of the dominant Nitrosopumilaceae clade (Eta clade) in the basement compared to the seawater. This study explored AOA and their activity in the upper oceanic crust, and our results have ecological implications for the biogeochemical cycling of nitrogen in the crustal subsurface.

**IMPORTANCE** Ridge flanks represent the major avenue of chemical and heat exchange between the Earth’s oceans and the lithosphere and are thought to harbor an enormous and understudied biosphere. However, little is known about the diversity and functionality of the crustal biosphere. Here, we report an indigenous community of archaea specialized in ammonia oxidation (i.e., AOA) in the oxic oceanic crust at North Pond. These AOA are the dominant archaea and are likely responsible for most of the cycling taking place in the first step of nitrification, a feasible nitrogen cycling step in the oxic basement. The crustal AOA community structure significantly differs from that in deep ocean water but is similar to that of the community in the overlying sediments in close proximity. This report links the occurrence of AOA to their metabolic activity in the oxic subseafloor crust and suggests that ecological selection and *in situ* proliferation may shape the microbial community structure in the rocky subsurface.

## Abstract (precis)

Ridge flanks represent the major avenue of chemical and heat exchange between the Earth’s oceans and the lithosphere and are thought to harbor an enormous and understudied biosphere. However, little is known about the diversity and functionality of the crustal biosphere. Here, we report an indigenous community of archaea specialized in ammonia oxidation (i.e., AOA) in the oxic oceanic crust at North Pond. These AOA are the dominant archaea and are likely responsible for most of the cycling taking place in the first step of nitrification, a feasible nitrogen cycling step in the oxic basement. The crustal AOA community structure significantly differs from that in deep ocean water but is similar to that of the community in the overlying sediments in close proximity. This report links the occurrence of AOA to their metabolic activity in the oxic subseafloor crust and suggests that ecological selection and *in situ* proliferation may shape the microbial community structure in the rocky subsurface.

## INTRODUCTION

The upper oceanic crust constitutes one of the largest ecosystems on Earth (1, 2). Most oceanic crust is buried under sediment; in young ridge flanks, however, it is geochemically well connected with the surface world (3–5). The young crust is highly permeable and functions as an aquifer system in which vast volumes of seawater continuously flush in and out (6). As a consequence, changes in seawater composition during the residence time (representing hundreds of thousands of years on average) influence ocean chemistry (7–10). Diverse microbes are ubiquitous in this vast habitat (as reviewed in references 11 and 12), their total biomass has been argued to exceed that of global marine sediments (13), and their activity is thought to exert a significant influence on the partitioning of chemical species between the subsurface and surface world.

Despite the potentially global importance of microbial life within the upper crust, fundamental questions regarding cellular recruitment, extent of activity, niche partitioning, and community succession in this habitat remain unanswered. There have been only a few published studies dedicated to microbial interrogation of native subseafloor crustal rocks (1, 14–17) and even fewer that have addressed the potential biogeochemical functions of these communities (16, 18). Based on the limited body of literature, it appears that microbial communities in the subseafloor crust are unique in comparison to those found in hydrothermal plumes, deep seawater, and hydrothermal fluids and in seafloor exposed basalt (12) and the overlying sediments (19). The nature of the ecological mechanism driving crustal community selection remains an open issue confounded by the technical difficulty of high-quality sample collections.

Scientific explorations at North Pond, a sediment-filled pond on the western flank of the Mid-Atlantic Ridge, have resulted in the successful retrieval of pristine sediment-buried oceanic crust and of the overlying sediment (20), as well as of bottom seawater (BS) (10, 21, 22). These samples provide an unprecedented opportunity to systematically evaluate the dispersal patterns and potential functions of microbes across benthic regimes. The geological settings on this ridge flank, i.e., low-temperature and oxic hydrothermal circulation conditions (21, 23), make North Pond a site that is representative of subsurface habitats beneath a wide range of ridge flanks globally (1, 20).

Previous studies at this site have revealed diverse microbial communities with abundances of up to ∼10^5^ cells g^−1^ rock in the igneous crust (15, 19, 24). In addition, microbial nitrogen transformation occurs in the overlying sediments from the sediment-water interface down to the basement, resulting in a downward flux of nitrate into the underlying oceanic crust (25). The presence of ammonia-oxidizing archaea (AOA) in the bathypelagic seawater (22, 26, 27), the subseafloor sediments (25), and the crust (19, 22) further suggests that microbial nitrogen transformations are pervasive in the deep biosphere at this site.

**FIG 1:**
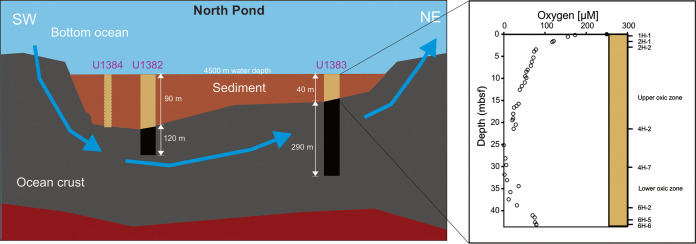
Locations of the deep habitats (bottom seawater, subseafloor sediments, and ocean crust) at North Pond. (Left) Schematic graph showing the sampling locations of the three sites studied in this study (modified from reference 35). The blue arrow denotes the direction of seawater circulation in the oceanic crust between the two sites. NE, northeast; SW, southwest. (Right) Depths and niches of sediment horizons (upper and lower oxic zones) selected from hole U1383E and the oxygen profile reported in reference 23.

In this study, we investigated the occurrence and community composition of AOA in basaltic rocks from two boreholes (U1383C and U1382A) collected from North Pond (19). To determine the origin and dispersal of the AOA community, we also surveyed AOA in one bottom seawater sample collected in 2014 (22) and the entire sediment column collected at hole 83E (25) (Fig. 1). For characterization of AOA communities at high taxonomic resolution, we used two phylogenetic markers, i.e., archaeal 16S rRNA and *amoA* genes, whose phylogenies are congruent down to at least the taxonomic rank of order (28, 29) and can be reliably used to distinguish environmentally relevant suborder clades (30). We also explored the possibility of *in situ* nitrification within the upper ocean crust using a hydrogeological box model (14) based on the basal sediment geochemistry.

## RESULTS

### Geochemical evidence for nitrate production in the oceanic crust.

**FIG 2:**
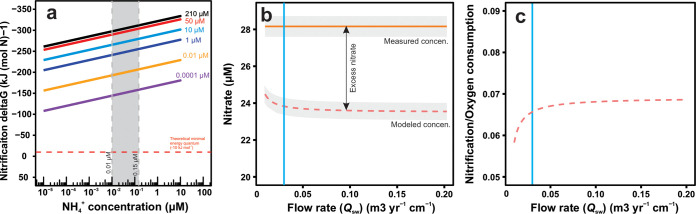
Gibbs free energy of nitrification (a), nitrate dynamics (b), and the ratio of nitrification to oxygen consumption (c) in the basement between sites U1384 and U1383. (a) Gibbs free energy levels calculated for complete nitrification (NH_4_^+^ + 2O_2_ → NO_3_^−^ + 2H^+^ + H_2_O) under conditions of a wide range of NH_4_^+^ concentrations (10^−5^ to 10 μM) and O_2_ concentrations (0.0001 to 210 μM). The gray band in panel a denotes the range of NH_4_^+^ concentrations measured in North Pond crustal fluids (0.01 to 0.15 μM). The red dashed line represents the theoretical minimal energy quantum of life of −10 kJ mol^−1^ (90). (b and c) Nitrate concentration (b) and nitrification/oxygen consumption ratio (c) in the formation fluids at U1383D were predicted and are depicted in red dashed lines, with the fluid flow rate in the range of 0.01 to 0.20 m^3^ year^−1 ^cm^−1^. The light orange horizontal line in panel b denotes the nitrate concentration measured at the sediment-basement interface of hole U1383D. Gray areas represent the uncertainty that stemmed from the 2% variation in nitrate concentration measurements. The vertical blue lines in panels b and c indicate the flow rates constrained on the basis of a previously reported Sr porewater profile (23).

Based on the thermodynamic calculation shown in Fig. 2A, nitrification is a highly favorable process that occurs in the presence of a wide range of combinations of O_2_ and NH_4_^+^ concentrations, including the conditions found in the crustal aquifer at North Pond (i.e., 210 μM O_2_ [21, 22] and 0.01 to 0.15 μM NH_4_^+^ [31]). Assuming that the chemical composition of circulating fluids in the crustal basement is in equilibrium with the sediment porewater at the sediment-basement interface, nitrate concentration in the formation fluids should be identical to that in the basal sediment porewater, i.e., 28.2 μM and 23.5 μM at sites 83 and 84, respectively (32). These values, with a 2% level of uncertainty (22), are significantly higher than the nitrate concentration measured in the bottom seawater (∼21.1 μM) (21, 22, 33), indicating an enrichment of nitrate in the basement. To explore whether the downward diffusive flux of nitrate from the overlying sediments (25) is sufficient to explain the nitrate enrichment, we applied a previously described box model (34), with the volumetric flow rate *Q*_sw_ in the previous constrained range of 0.01 to 0.2 m^3^ year^−1 ^cm^−1^ (23), to study the dynamics of nitrate in the flow path between boreholes 84A and 83D. Results showed that, under conditions in which the nitrate concentration is not influenced by any process in the basement, the level of nitrate in the formation fluids at hole 83D should range between 23.5 and 24.0 μM (Fig. 2B). These values are ∼4 μM lower than the concentration expected from the porewater profile (28.2 μM), indicating that other processes may produce nitrate (e.g., nitrification) within the basaltic crust (Fig. 2B). Furthermore, assuming the same reaction zone depth and porosity of the upper oceanic crust as previously reported (23), we estimated the net nitrification rate to account for 6% to 7% of crustal oxygen consumption in the flow path from hole 84A to hole 83D (Fig. 2C).

### Quantification of ammonium oxidizers in the subseafloor basalts.

**FIG 3:**
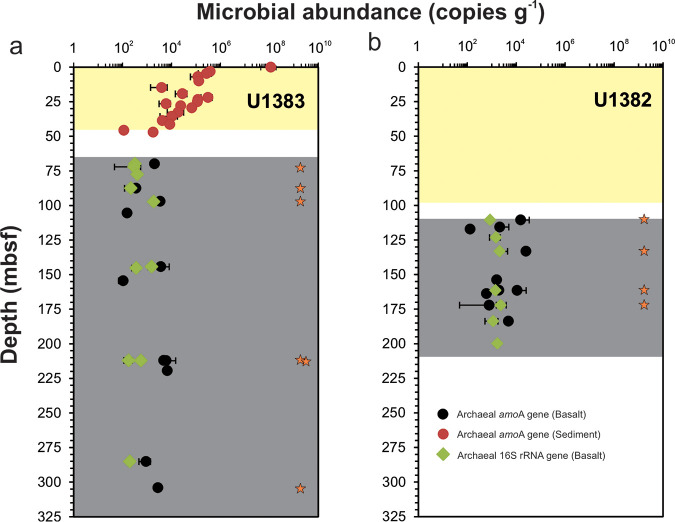
Abundances of archaeal *amoA* and 16S rRNA genes in sediments and basaltic rocks at sites U1383 (a) and U1382 (b) beneath North Pond. In panels a and b, light yellow zones indicate the sediment column, while light gray zones denote basaltic crust. Gaps between the two zones represent sequences not recovered. Stars mark the basaltic rocks from which archaeal *amoA* genes were successfully amplified and sequenced. Error bars represent standard deviations of results from triplicate quantitative PCR (qPCR) measurements. Archaeal *amoA* gene abundance data are from reference 25.

The first step of nitrification, ammonia oxidation, can be performed by ammonia-oxidizing archaea (AOA) and bacteria (AOB). We enumerated both groups by quantitative PCR targeting their functional *amoA* genes. Whereas archaeal *amoA* genes were detected in most (21 of 33) of the basaltic rocks (Fig. 3), the bacterial counterparts were undetectable. In hole 82A, the levels of AOA *amoA* genes were above the detection limit (∼150 copies g^−1^ rock) in 10 of the 16 samples, differing in the range of 1.32 × 10^2^ to 1.54 × 10^4^ copies g^−1^ rock (Fig. 3b). These *amoA* gene abundances should represent the AOA cell abundances, because all known AOA harbor a single *amoA* gene copy (30). The highest abundance was found at ∼133 meters below the seafloor (mbsf) (sample 82A_5R_1B), a depth characterized by strongly altered hyaloclastite with a high vein abundance (35). At site 83C, archaeal *amoA* genes were detected in 11 of the 17 samples at levels ranging from 1 × 10^2^ to 6.8 × 10^3^ copies g^−1^ rock (Fig. 3a). The highest abundance was observed at the interval of ∼212 to 220 mbsf (samples between 19R and 20R), and those results also exhibited the highest degree of alteration (in volume of alteration halos) (36). Compared to the overlying sediments at hole 83E, archaeal *amoA* gene abundances were found to be in the same order of magnitude as the basal part of the overlying sediment column (∼10^3^ copies g^−1^ sediment) but significantly lower than in the surface sediments (∼10^8^ copies g^−1^ sediment) (Fig. 3a). We failed to detect copies of the *nxrB* gene, encoding the nitrite oxidoreductase beta subunit in nitrite-oxidizing bacteria (NOB) that is responsible for the second step of nitrification, in any of the basaltic rocks, although they were successfully detected in the overlying sediments using the same protocol (25).

### Relative abundances of nitrifiers.

**FIG 4:**
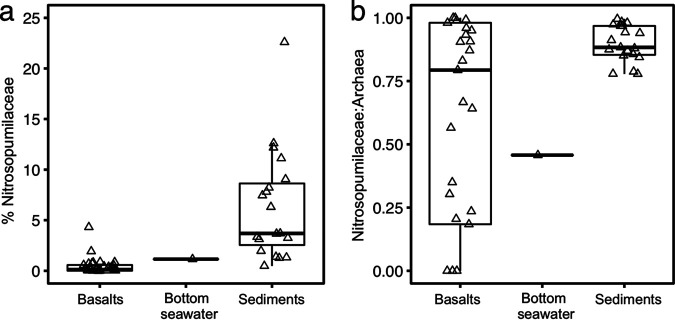
Box plots of relative abundances of Nitrosopumilaceae in the total community (a) and the archaeal community (b) in the North Pond bottom seawater, sediments, and basalts. These relative abundances were assessed by the 16S rRNA amplicon sequencing.

In the 16S rRNA gene amplicon sequencing data, putative AOA of the archaeal family of Nitrosopumilaceae were detected in 78% of the basaltic rocks (26 of 33), while AOB and NOB were detected only occasionally (7 of 33 for AOB and 17 of 33 for NOB) (see Table S1 in the supplemental material). Nitrosopumilaceae accounted for a smaller fraction of the total prokaryotic community in the basalts (0.4% ± 0.9% [means ± standard deviations {SD}]) than in the subseafloor sediments (6.5% ± 5.9%) and bottom seawater (1.15%) (Fig. 4a). Based on the Nitrosopumilaceae/Archaea ratio, Nitrosopumilaceae was the dominant archaeal taxon in the basaltic rocks (65% ± 38%, *n *= 33), subseafloor sediments (89% ± 7%, *n *= 19), and bottom seawater (46%, *n *= 1) (Fig. 4b).

10.1128/mSystems.00758-19.3 TABLE S1 Proportion of ammonia-oxidizing bacteria (AOB) and nitrite-oxidizing bacteria (NOB) assessed by 16S rRNA gene amplicon sequencing. Download Table S1, DOCX file, 0.02 MB. Copyright © 2020 Zhao et al. 2020 Zhao et al. This content is distributed under the terms of the Creative Commons Attribution 4.0 International license.

### Diversity and richness of AOA at North Pond.

Nitrosopumilaceae 16S rRNA gene sequences from the 29 samples (9 rock samples, 19 sediment samples, and 1 bottom seawater sample) were clustered into 38 operational taxonomic unites (OTUs), whereas the 602 AOA *amoA* sequences recovered from the 19 samples (10 rocks, 8 sediments, and 1 bottom seawater) were grouped into 15 OTUs (Table 1). Although only a limited number (≤43) of *amoA* gene sequences were obtained from each sample, the observed richness (*S*_obs_) was identical to the estimated richness (*S*_chao1_), except for the levels seen with the two uppermost samples in the upper oxic sediments (Table 1), suggesting that our *amoA* gene sequencing efforts captured most of the diversity within the Nitrosopumilaceae community, as also inferred from the high levels of library coverage (89% to 100%) (Table 1) and plateau rarefaction curves (Fig. S1).

**TABLE 1:** Diversity measures of Nitrosopumilaceae community in the three deep habitats at North Pond

Habitat	Horizon	Depth (mbsf)	16S rRNA gene amplicon sequencing					Archaeal *amoA* gene clone library			
			No. of reads after filtering^*a*^	Total no. of OTUs^*a*^	No. of Nitrosopumilaceae OTUs	Total no. of Nitrosopumilaceae reads	% Nitrosopumilaceae	No. of clones	Observed richness (no. of OTUs)	Estimated richness (no. of OTUs)	% coverage
Bottom seawater		0	46,496	654	9	520	1.15	36	3	3	100

Sediment	1H-1	0.1	31,478	1,046	31	7,426	23.48	36	8	14	89
	2H-1	3.1	23,795	456	23	3,368	14.14	33	6	7	94
	2H-2	4.6	20,836	461	27	2,878	13.80	43	7	7	95
	4H-2	22.0	32,533	416	27	3,349	10.28	38	7	7	97
	4H-7	29.5	25,830	178	7	1,000	3.87	17	1	1	100
	6H-2	41.4	23,972	145	4	733	3.06	36	1	1	100
	6H-5	45.7	39,019	223	5	337	0.86	36	3	3	97
	6H-6	47.0	26,401	224	6	296	1.12	26	2	2	100

U1383C basalt	2R_2E	72.2	19,935	334	2	59	0.30	38	2	2	100
	4R_1B	87.6	18,980	135	2	134	0.71	32	1	1	100
	5R_1B_I	97.0	15,507	194	3	299	1.93	39	1	1	100
	19R_1B	212.2	19,538	280	1	48	0.25	37	1	1	100
	19R_1A	212.0	18,955	142	0			11	1	1	100
	30R_1A	304.0	22,851	235	1	7	0.03	10	1	1	100

U1382A basalt	2R_1C	110.6	8,705	397	3	50	0.57	38	1	1	100
	5R_1B	133.2	20,777	293	4	178	0.86	35	1	1	100
	6R_1A	142.4	22,324	187	1	73	0.33	30	2	2	100
	9R_1C	171.2	17,744	364	2	135	0.76	31	1	1	100

a Data are from Jørgensen and Zhao (19).

10.1128/mSystems.00758-19.1 FIG S1 Rarefaction curves of archaeal *amoA* gene phylotype richness in clone libraries of the bottom seawater (blue line), subseafloor sediments (light yellow lines), and basaltic rocks (black lines) beneath North Pond. OTUs were defined using a cutoff of 95% *amoA* gene nucleotide sequence identity. Download FIG S1, PDF file, 0.4 MB. Copyright © 2020 Zhao et al. 2020 Zhao et al. This content is distributed under the terms of the Creative Commons Attribution 4.0 International license.

Analyses based on both phylogenetic markers showed that the level of Nitrosopumilaceae richness was lower in the basaltic rocks than in the bottom seawater: maximum levels of 4 Nitrosopumilaceae 16S rRNA OTUs and 2 *amoA* gene OTUs were observed in individual basalt samples, while 9 Nitrosopumilaceae 16S rRNA gene OTUs and 3 *amoA* gene OTUs were observed in the bottom seawater (Table 1). Compared to the overlying sediments, the richness in the basalt was similar to that found at the basal sediments (4 to 6 Nitrosopumilaceae 16S rRNA gene OTUs and 1 to 3 *amoA* gene OTUs in individual horizons) but significantly lower than that observed in the surface sediments (23 to 31 Nitrosopumilaceae 16S rRNA OTUs and 6 to 8 *amoA* OTUs).

### Phylogeny and community structure of AOA at North Pond.

**FIG 5:**
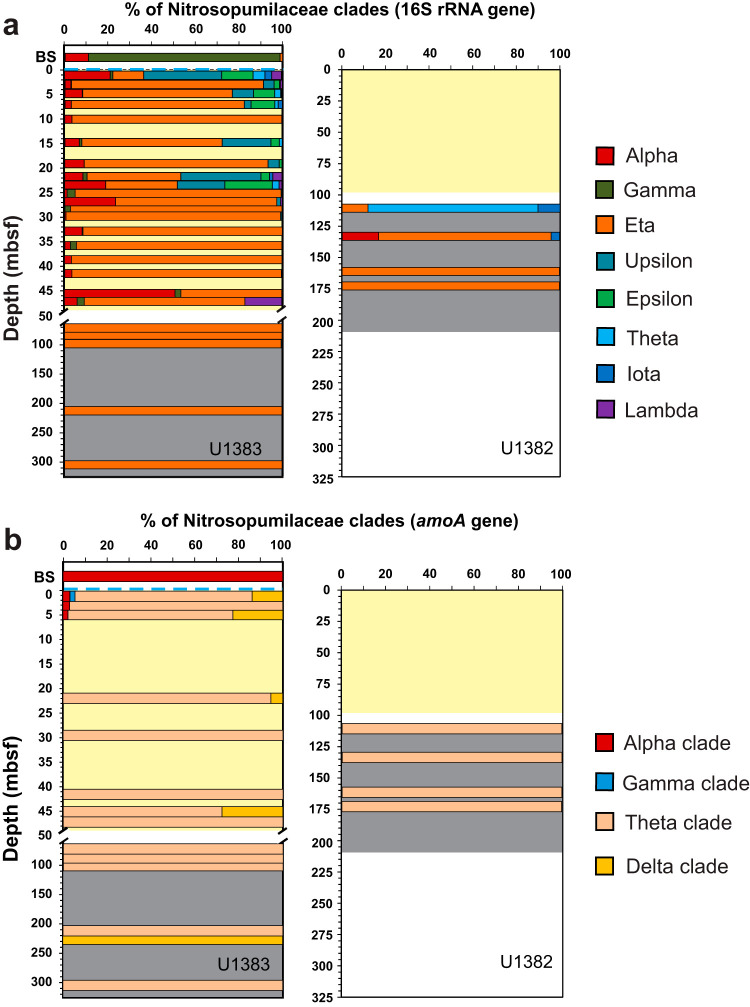
Community structure of Nitrosopumilaceae in the bottom seawater (BS), subseafloor sediments, and basaltic rocks at North Pond, assessed by the phylogenetic maker of the 16S rRNA gene (a) and the functional *amoA* gene (b). Dashed blue lines represent the sediment-water interface. Light yellow denotes the sediment column, while gray represents the basaltic crust. OTUs of both genes were grouped into clades according to their phylogeny as shown in the phylogenetic trees.

**FIG 6:**
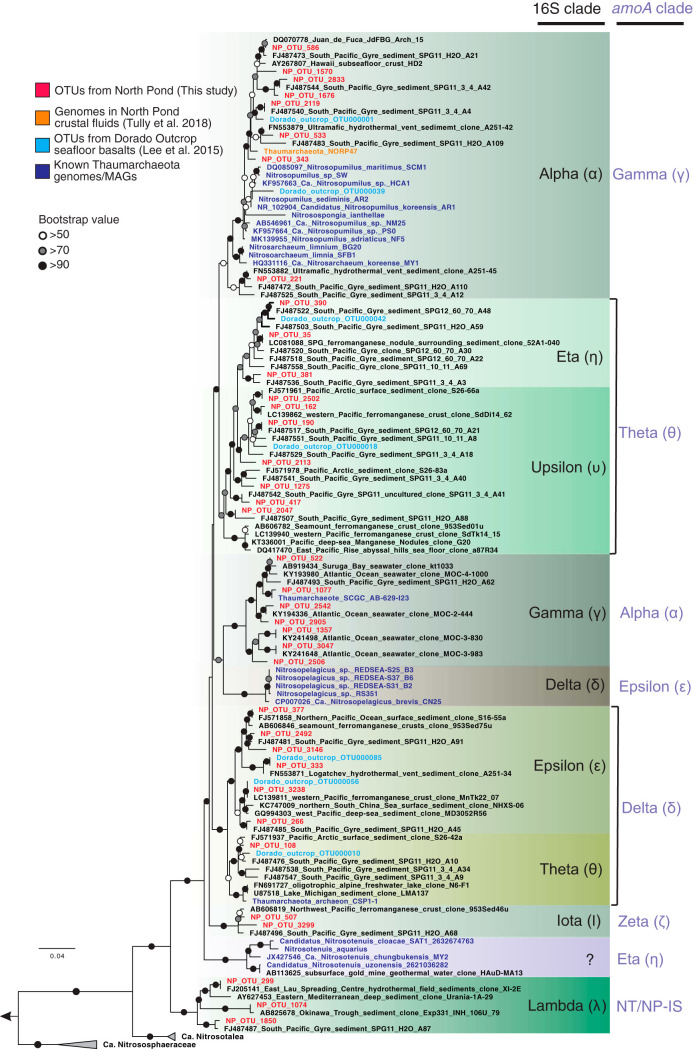
Maximum likelihood phylogenetic tree of the 16S rRNA gene sequences of Nitrospumilaceae. The tree was constructed using a maximum likelihood algorithm with IQ-TREE and the best-fit GTR+F+I+G4 model. The tree is rooted to two thermophilic Thaumarchaeota species (*Candidatus* “Nitrosocaldus cavascurensis” SCU2 and *Candidatus* “Nitrosocaldus islandicus”). Representatives of OTUs recovered from the three deep habitats beneath North Pond are shown in bold red. Bootstrap values (1,000 replicates) supporting the nodes are indicated by filled circles as follows: black, >90%; gray, >70%; white, >50%. The scale bar indicates estimated substitutions per site. Nomenclature of clades follows that used in references 79 and 81, whereas data representing one clade harboring newly characterized AOA are indicated with question marks. Also shown on the right side are the corresponding clades (light blue) of *amoA* genes.

Phylogenetic analysis of the 38 Nitrosopumilaceae 16S rRNA gene OTUs showed that the Eta (η) clade dominated the basaltic crust (Fig. 5a), with the exception of the shallow basalt in hole 82C, where the Theta (θ) clade was most abundant. The Eta clade was also dominant in the subseafloor sediments (72% on average), in which the Alpha (α; 11% on average) and Upsilon (υ; 9% on average) clades were also detected (Fig. 5a). Sequences in the Eta clade are related only distantly to Nitrosopumilaceae isolates or enriched cultures and show high similarity to uncultured environmental sequences collected exclusively from pelagic sediments (Fig. 6). In contrast, the bottom seawater was mainly composed of representatives from the Gamma (γ; 88%) and Alpha clades (11%), while the Eta clade was found to be less abundant (1.15%) (Fig. 5a). Additional, minor clades were observed mainly in the upper oxic sediments in hole 83E. Examining the Nitrosopumilaceae community structure at the individual OTU level, the most dominant clade in the basalts (OTU35, Eta clade) was also detected in the bottom seawater, albeit at a lower relative abundance (see Fig. 8a).

To check if the phylotypes detected at North Pond basalts are also present at other sites, we analyzed the Nitrosopumilaceae communities in the seafloor basalts at Dorado Outcrop (37), another oxic and young ridge flank system on the eastern flank of the East Pacific Rise (38). We found that the Eta clade (represented by Dorado_otucroo_OTU000042) (Fig. 6) existed in all 14 examined basalts and accounted for 5.2% of the Nitrosopumilaceae communities on average (see Fig. S2 in the supplemental material), whereas the overall levels of the Nitrosopumilaceae communities at the Dorado Outcrop basalts were similar to those in the local Pacific Ocean bottom seawater and were dominated by the Alpha clade (74.1% on average) (Fig. S2).

10.1128/mSystems.00758-19.2 FIG S2 Distribution of Nitrosopumilaceae 16S rRNA gene OTUs in the seafloor basalts at Dorado Outcrop. The clade affiliations of these OTUs were determined based on their placement in the phylogenetic tree of 16S rRNA gene shown in Fig. 6. “BW1” and “BW2” denote the two bottom seawater samples, while the rest are the seafloor basalts of various degrees of alteration. This figure was made based on data published in reference 36. Download FIG S2, PDF file, 0.4 MB. Copyright © 2020 Zhao et al. 2020 Zhao et al. This content is distributed under the terms of the Creative Commons Attribution 4.0 International license.

**FIG 7:**
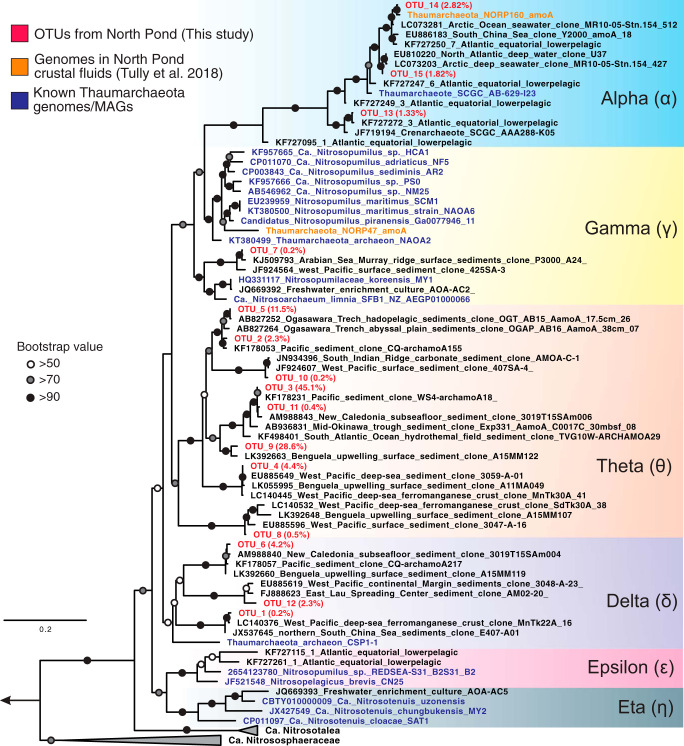
Maximum likelihood phylogenetic tree of the archaeal *amoA* gene. The tree was reconstructed using IQ-TREE with GTR+F+I+G4 as the best-fit evolutionary model and was based on an alignment of 592 nucleotide positions. The tree is rooted to two thermophilic *Thaumarchaeota* (*Candidatus* “Nitrosocaldus cavascurensis” SCU2 and *Candidatus* “Nitrosocaldus islandicus”). Representatives of OTUs recovered from the three deep habitats beneath North Pond are shown in bold red, and their relative abundances in the whole data set are indicated in the brackets. Bootstrap values (1,000 replicates) supporting the nodes are indicated by filled circles as follows: black, >90%; gray, >70%; white, >50%. The scale bar indicates estimated substitutions per site. Nomenclature of clades follows reference 30.

**FIG 8:**
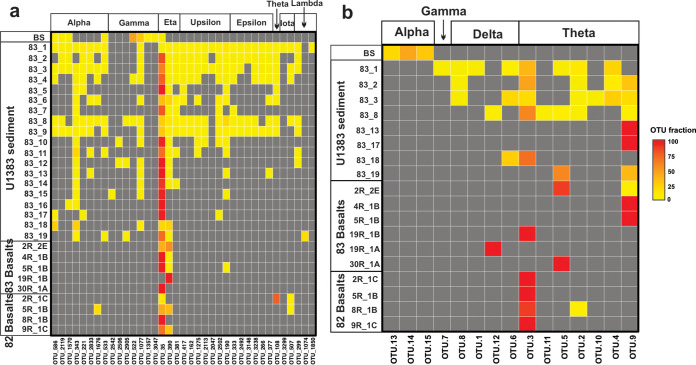
Heat map of the Nitrosopumilaceae OTUs of the 16S rRNA gene (a) and the *amoA* gene (b). The clades are indicated at the top. Note that the clades of these two maps denoted by the Greek letters do not correspond to each other. Data representing the 16S rRNA gene in 83E are from reference 25. BS, bottom seawater.

The archaeal *amoA* gene sequences recovered from the North Pond basalts and sediments were found to be primarily affiliated with the Theta (θ) clade (Fig. 5b), comprised of seven OTUs (OTU2, OTU3, OTU4, OTU5, OTU9, OTU10, and OTU11; see Fig. 8b), while all sequences recovered from the bottom seawater (including OTU13, OTU14, and OTU15) were placed into the Alpha (α) clade (Fig. 5b; see also Fig. 8b). Similarly to the 16S rRNA gene sequences, *amoA* gene sequences from the predominant Theta clade were found to be distinct from all AOA isolates or enriched cultures and showed high similarities to uncultured AOA sequences from oligotrophic marine sediments beneath pelagic oceans, including the South Atlantic Gyre (39), the West Pacific Ocean (40), and the New Caledonia Basin (41) (Fig. 7). The remaining *amoA* OTUs, mainly detected in the upper sediment layers in 83E, were assigned into the Delta (δ) clade (including OTU1, OTU6, and OTU12), which also lacks cultured representatives (Fig. 7). Notably, at the OTU level, the members of the Theta clade (7 OTUs), which are dominant in the basalts and basal sediments, were not detected in the bottom seawater (Fig. 8b), probably due to the low sequencing depth of the *amoA* gene clone libraries (<43 sequences per sample).

## DISCUSSION

Microbes are ubiquitous in the rocky subsurface, with their metabolic functions largely uncharted. A number of potential geochemical processes likely to sustain the deep crustal biosphere have been suggested, including the oxidation of sulfur (42), iron (15, 42), and hydrogen (43) and the reduction of sulfate (16). In this study, we focused on elucidating the occurrence and ecological functions of archaea, an understudied domain of life, in the oxic subseafloor ocean crust at North Pond. Although the geochemical modeling suggests the feasibility of complete nitrification, we were able to detect only the AOA community responsible for the first step of nitrification, in most of the basaltic rocks. The presumed low abundances of NOBs in these basalts likely contribute to their inconsistent detection in our survey. AOA lineages account for <1% of the total prokaryotic communities; however, they are the dominant archaea and may thus play an important role in nitrification in the oxic subseafloor oceanic crust. We discuss the activity, diversity, and origin of AOA in the oxic oceanic crust at North Pond.

### Evidence for *in situ* nitrification in the upper oceanic crust.

The chemical and physical characteristics of North Pond make it representative of many ridge flank ecosystems, as delineated by volcanic crustal age, fluid temperature, and extent of water-rock interaction (1). At this site, we noted nitrate enrichment in the crustal fluids at the sediment/basement interface relative to background seawater. In line with its representation, this geochemical signal has been detected in the few studied locations characterized by vigorous low-temperature hydrothermal circulation (e.g., the East Pacific Rise [44] and the Clarion-Clipperton Fracture Zone [45]). Such nitrate enrichment in the crust may result from (i) high-level downward nitrate flux from the overlying sediments (25) or (ii) a downward flux combined with *in situ* nitrification.

We then explored the nitrification potential in the North Pond basement using a reaction-transport model that was previously applied to estimate the oxygen consumption rates in the same crustal setting (23). Our modeling suggested that nitrification is thermodynamic favorable and, indeed, needed to account for the excess nitrate in the basement (Fig. 2). Due to the low biomass (15, 19) and sluggish reaction rates (23) in such an oligotrophic environment, the signal of excess nitrate is weak (∼4 μM) but is distinguishable from the standard deviation (2%) (22) of nitrate measurements and is helpful to evident the nitrification process and complement our molecular assessments. Nitrification is the only likely source of this excess nitrate and fuel nitrifiers within the upper oceanic crust (Fig. 2). While analyses of the exact volumetric reaction rates of nitrification would provide valuable information, the lack of a nitrate profile representative of depths deeper than the sediment/basement interface constrains our model output to two dimensions. Nevertheless, considering the presence of ammonia-oxidizing Thaumarchaeota genomes reported in the crustal fluids at this site (22), it is likely that nitrification—at least, ammonia oxidation—is an important biogeochemical process in the upper oceanic crust at North Pond.

We note that the recently reported nitrate concentrations measured in the pristine crustal fluids (21.1 to 22.3 μM) (22), collected from a depth interval spanning almost 76 m (from 70 to 146 m below the sediment-basement interface), are only slightly higher than the bottom seawater concentration (21.05 ± 0.49) (33) and lower than what we use in our model. However, considering the ubiquitous denitrification potential suggested by the presence of reconstructed denitrifying bacterial genomes in these fluids (22), the crustal fluid nitrate would be consumed by denitrifiers and the concentration would reach a level lower than those measured in the bottom seawater if nitrification were not occurring there to restore it. Therefore, the seawater-like nitrate concentrations measured in the crustal fluids still point to a nitrification potential in the basement beneath North Pond. The idea of the occurrence of nitrification in the North Pond basement was also supported by the increase of fluid nitrate concentration measurements over the course of 7 years after drilling (31).

Although the measured NH_4_^+^ concentrations in the crustal fluids at North Pond were extremely low (0.01 to 0.15 μM), the microbial degradation of refractory organic matter in the crust suggested by the organic matter concentration and isotope data (10) could have provided ammonium to the AOA community, at least to some extent. This supposition is supported by our calculation estimating that nitrification accounts for 6% to 7% of oxygen consumption at North Pond. These values are close to the nitrification/oxygen consumption ratio (∼9.4%) in oxic marine sediments (see, e.g., references [Bibr B46][Bibr B47][Bibr B48]) where ammonium liberated from the aerobic mineralization of organic matter is completely oxidized to nitrate by aerobic nitrifiers. The existence of putative anaerobic microbes, e.g., sulfate reducers in the basement rocks (19) and fluids (22), probably promoted by the presence of particle microenvironments under bulk oxic conditions (49), could explain why the relatively minimally abundant nitrifiers account for 6% to 7% of the O_2_ consumption of the total communities.

### Presence of indigenous ammonia oxidizers in North Pond basalts.

The idea of active nitrification in the basement, as inferred by the geochemical modeling, was supported by the molecular survey confirming the presence of putative nitrifiers. The first step of nitrification, ammonia oxidation, in the oceanic crust at North Pond is likely mediated primarily by Archaea affiliated with Nitrosopumilaceae, due to the following observations: (i) 16S rRNA gene sequences affiliated with known AOB genera were detected only occasionally (7 of the 33 basalts, with ≤0.24% of total reads in individual basalt samples) (Table S1); (ii) the amplification of AOB *amoA* gene was unsuccessful; (iii) 16S rRNA gene sequences affiliated with known comammox *Nitrospira* species (50, 51) were not detected in any basalt. However, as AOB in laboratory cultures are known to have higher maximum per-cell activity than AOA (52), further experimental approaches like stable-isotope probing and the use of specific inhibitors are required to disentangle the roles of AOA and AOB in ammonia oxidation. The occasionally detected NOB are assumed to mediate the second step of nitrification. Their higher cell-specific catabolic rates (53) and higher biomass yield and growth rates (54) may enable them to keep pace with AOA and may explain the discrepancy in abundance levels between AOA and NOB. Despite being the dominant archaeal lineage in the basalts, Nitrosopumilaceae were successfully detected by both gene markers only in rocks with high biomass and with relatively high levels of alteration, suggesting a positive relationship between the stage of crustal alteration and the biomass that it supports, likely as a consequence of localized higher fluid flow (55).

Nitrosopumilaceae are the dominant archaea in the investigated basalts as shown by the high Nitrosopumilaceae/Archaea ratios (Fig. 4b). Nitrosopumilaceae also dominate archaeal communities in deep bathypelagic seawaters (26, 56), marine oxic sediments (25, 57), seafloor basalts (37), and ferromanganese nodules (58). However, Nitrosopumilaceae seem to be absent in anoxic and high-temperature ocean curst (16, 59, 60). Therefore, Nitrosopumilaceae archaea are probably widespread only in oxic oligotrophic habitats and influence the nitrogen cycling there.

The prevalence of AOA in surrounding environments (seawater and oxic sediments) and the inherent low biomass in the basaltic rocks warrant caution in interpreting molecular evidence due to the risk of contamination (19, 61–63). However, a number of observations suggest that our AOA sequences do not represent contaminants. First, Nitrosopumilaceae are not found in DNA extraction kits and PCR reagents (64), as independently confirmed by our analyses of the experimental controls (i.e., the two blank extractions), in which no Nitrosopumilaceae sequences were amplified. Second, phylogenetic analyses at the OTU level showed that the dominant AOA clades in the subsurface rocks are markedly different from those in the bottom seawater, which, along with the distinct community structure, suggested little to no seawater contamination (Fig. 5). These observations strongly suggest that our results did not arise from the presence of contamination, crust-preserved necromass, relic nucleic acids in circulating seawater, and/or transient archaeal cells.

### Origin, diversity, and potential for growth of Nitrosopumilaceae in oceanic crust.

The origin of microbial life found in subsurface oceanic crust has been debated, but seeding from the circulating seawater seems to be the prevailing idea (11, 65). We found that Nitrosopimulaceae were also prevalent in the bottom seawater sample, where they comprised ∼50% of the archaeal community (Fig. 4b). Although only one bottom seawater sample was analyzed here, the Nitrosopimulaceae community that we detected in it could well represent the deep ocean Thaumarchaeota community at North Pond, for the following reasons: (i) the AOA community resembles those previously reported in deep bathypelagic waters in North Atlantic Gyre (66) and (ii) Thaumarchaeota community composition variation is limited in lower bathypelagic depths (27). The most dominant Nitrosopumilaceae OTU in North Pond basalts (OTU35) was also present in the bottom seawater, albeit at a much lower relative abundance (Fig. 8a), suggesting that it could have been seeded from the seawater entering the aquifer system. However, such an observation could also be explained by cell export from the crustal aquifer to the overlying ocean. Irrespective of the direction of dispersal, hydrothermal circulation not only can facilitate the exchange of chemical constituents (7) but also can link the microbial communities between the deep ocean and oceanic crust.

Physical separation between the bottom seawater and the sediment-buried basaltic crust caused the long residence time of circulating fluids and resulted in the partitioning of the AOA communities in these two environments at North Pond (Fig. 6 and 8). That this phenomenon was not observed at Dorado Outcrop (see Fig. S2 in the supplemental material) was probably due to the direct contact between the two habitats. We propose that both environmental selection and *in situ* microbial growth may shape the AOA community structure in the oceanic crust at North Pond. The crustal environment may select against the clades dominating in the bottom seawater (the Alpha and Gamma clades of 16S rRNA gene or the Alpha clade of *amoA* gene), because these clades were undetectable in any of the basalt rocks. Likewise, the crustal environment may select for the colonization of the subsurface clade (the Eta clade of the 16S rRNA gene or the Theta clade of the *amoA* gene), as evidenced by its enrichment in the oceanic crust. To elucidate whether the enrichment of the Eta clade resulted from passive retention or from *in situ* growth, we calculated and compared the abundances of the members of Eta clade between the bottom seawater and basalt rocks. On the basis of previous reported cell abundances (2.2 × 10^4^ cell/ml) (21) and our amplicon sequencing data (1.15% of total archaeal community), the number of Nitrosopumilaceae Eta clade cells in bottom seawater per cubic centimeter can be estimated to be 20. The corresponding number in the basaltic crust, considering the average porosity of 4% and assuming a density of 3 g cm^−3^ for habitable pore space, is in the range of 7.5 × 10^3^ to 750 × 10^3^ AOA cells cm^−3^. The apparently higher abundance of Nitrosopumilaceae Eta clade cells in the crustal fluids than in bottom seawater suggested that these cells are not transient but are instead enriched in the basement, perhaps due to *in situ* growth. The measured autotrophic and heterotrophic microbial activities in these boreholes (21) may provide clues regarding the energetics that could support *in situ* microbial growth.

A high degree of similarity in terms of richness, diversity, community composition, and community structure was observed between the Nitrosopumilaceae community in the basal sediment and the buried basaltic crust (Table 1) (Fig. 4, 5, and 6). In particular, both environments seem to host low diversity of AOA, dominated by the Nitrosopumilaceae Eta clade. A similar finding was reported from comparisons between the deep sediment horizons and subsurface crustal fluids at the Juan de Fuca Ridge (67). Considering the known chemical exchange between these two habitats (see, e.g., references 23, 34, 45, and 68), it is tempting to assume that microbial dispersal across the sediment-basement interface is possible and could drive Nitrosopumilaceae community similarities across these two subbenthic habitats. However, this scenario would require active cell migration across the sediment-basement interface, which is thought to be an extremely energy-consuming process (69). Furthermore, there is no vertically decreasing trend with depth observed for the Nitrosopumilaceae abundance in either of the two boreholes, also making microbial dispersal (basal sediment to crust) less likely as the primary source of crustal Nitrosopumilaceae. Therefore, we speculate that similar environmental settings (e.g., oxic environments and low ammonium concentrations) may drive the selection of similar Nitrosopumilaceae assemblages in these two habitats.

The dominant Nitrosopumilaceae clades (Eta, Upsilon, and Theta clades of 16S rRNA gene) detected in North Pond subseafloor basalts are also present in the seafloor basalts at Dorado Outcrop (37). The much higher percentages (∼15% of the total community) and seawater-like community structure of Nitrosopumilaceae in the seafloor basalts at Dorado Outcrop likely result from direct contact with local bottom seawater. The prevalence of these clades in both the seafloor basalts at Dorado Outcrop and the subseafloor basalts at North Pond suggests that Nitrosopumilaceae clades are well adapted to the rock-attached lifestyles.

### Conclusion.

We explored the diversity and activity of AOA in a sediment-buried basaltic crust on an oxic and low-temperature ridge flank. Nitrification in the crust was thermodynamically favorable, and ongoing nitrification was inferred by application of a hydrological reaction-transport model. Phylogenetic analysis based on data representing 16S rRNA and archaeal *amoA* genes demonstrated that AOA communities in the basaltic crust are dominated by an uncharacterized Nitrosopumilaceae Eta clade. This clade is also prevalent in the overlying sediments and the seafloor basalts at Dorado Outcrop, suggesting that these AOA are well adapted to the oxic and oligotrophic habitats beneath pelagic oceans. Although dispersal of AOA across the sediment-basement interface cannot be completely ruled out, AOA most likely originate from the circulating seawater and are subsequently selected by rocky subsurface conditions, resulting in a subsurface-specific community. In sum, we show that archaeal communities in the oxic subseafloor oceanic crust at North Pond are dominated by the Nitrosopumilaceae lineage and that this lineage may play an active role in ammonia oxidation. Further experimental approaches such as incubations of isotope-labeled substrates (21, 57) are needed to confirm the dominant lineages, activity, and growth of AOA in this understudied ecosystem. While this study investigated a single location, microbiological and geochemical examinations of other ridge flank ecosystems are warranted for more insights into AOA and their ecological roles in the marine deep biosphere.

## MATERIALS AND METHODS

### Sample collection.

The basaltic rocks and sediments used in this study were retrieved during International Ocean Drilling Program (IODP) Expedition 336 from site U1382, hole A (82A), and site U1383, holes C and E (83C and 83E, respectively) (Fig. 1). The collection of sediment and basalt and descriptions of the samples were reported previously (19, 32). The bottom seawater analyzed in this study was collected 100 m above the seafloor by the use of a CTD (conductivity, temperature, and depth) instrument during a later cruise to North Pond (22). A 15-liter volume of seawater was filtered through a 0.2-μm-pore-size filter and preserved at –20°C for later analyses. Analysis was performed with a total of 53 samples from the following sources: 33 samples from basaltic rocks (17 from hole 83C and 16 from the nearby hole 82A), 19 samples from sediment horizons (hole 83E), and 1 bottom seawater (BS) sample. A complete sample list can be found in Table S1 in the supplemental material. In addition, we used the geochemical data retrieved from site U1384, hole A (84A), and from site U1383, hole D (83D), reported previously in reference 32, in a reaction-transport model to constrain the activities of nitrifiers (see below).

### Nitrate reaction modeling in the upper ocean crust.

We used a reaction-transport box model (34) to constrain the nitrate dynamics in the flow paths between different boreholes on the basis of the porewater profiles and downward fluxes of nitrate (25) in the basal part of each sediment core. A similar approach was employed previously by Orcutt et al. (23) to estimate the oxygen consumption rate in the upper ocean crust at North Pond. The nitrate concentrations that were present in the bottom seawater after drilling were determined to be rather stable (21.1 μM in 2012 [21] and 2014 [22]) using a continuous flow injection analyzer with an uncertainty level of ∼2% (21), consistent with the concentrations measured before drilling (21.05 ± 0.49 μM, *n *= 29) (33). Nitrate concentrations in the basal sediments were measured only one time during IODP Expedition 336 using the same method (70). Given that an 8.8-meter gap between the sediment/basement interface and the hydrological basement in hole 82B had been noted previously (32), nitrate concentrations in basement fluids at this site cannot be inferred from the basal sediments. Thus, only the flow path from site 84 to 83 was included in this study. The fluid flowing between these two drilling sites has previously been suggested to move in a northeasterly (NE) manner from site U1384 toward site 1383 (Fig. 1), based on the relative changes in basement fluid concentrations of strontium (Sr) (23). The model considers the scale defined by the distance between the two sediment holes (3.9 km between hole 84A and hole 83D), with a cross-section of 1 cm by 1 cm. The basement concentrations at the box terminus were determined for the geochemically conservative solutes used in the box model, assuming only diffusive removal/input across the sediment-basement interface and advective transport along the flow path. In contrast, for the geochemically active constituents (e.g., O_2_ and NO_3_^−^), differences between the model-calculated concentrations and those expected from linear extrapolation of the sediment pore water measurements were assigned to reactive processes within the basement, where the rate of nitrate reaction (*R*, in micromoles per year per centimeter) is calculated as follows:

$$R=\left({\left[{\text{NO}}_{\text{3}}{}^{-}\right]}_{\text{end}}\times Q_{\text{sw}}–{\left[{\text{NO}}_{\text{3}}{}^{-}\right]}_{\text{start}}\times Q_{\text{sw}}+F_{\text{d}}\right)/X$$

where [NO_3_^−^]_start_ and [NO_3_^−^]_end_ are the basement nitrate concentration at the start and end points of the box, respectively; *Q*_sw_ equals the volumetric flux of fluid within the basement (in cubic meters per year per centimeter), constrained by the diffusion behavior of the conservative solute Sr in the basement fluid as described previously (23); *F*_d_ is the diffusive flux of nitrate from sediment into basement perpendicular to the flow path, calculated to be 0.010 and 0.011 mmol m^−2^ year^−1^, in hole 84A and hole 83D, respectively (25), using Fick’s first law of diffusion (71); and *X* represents the distance between the start and end points (that is, between hole 84A and hole 83D). Under the same assumptions about the porosity and depth in North Pond as were described previously (23), and assuming 2 mol O_2_ (1.5 mol used in ammonia oxidation and 0.5 mol O_2_ in nitrite oxidation) are consumed in the nitrification process for each mole of NH_4_^+^, the ratio of the reaction rates of nitrification to oxygen respiration was calculated by dividing the nitrification rate by the oxygen respiration rates reported previously (23).

### Thermodynamic calculation of nitrification.

Gibbs free energy of nitrification (NH_4_^+^ + 2O_2_ → NO_3_^−^ + 2 H_2_O) was calculated as described previously (72). Briefly, data representing the standard Gibbs free energy of all reactants were retrieved and corrected to the *in situ* temperature (1.5°C) and pressure by the use of the R package *CHNOSZ* (73). To explore the feasibility of this process in the ocean crust beneath North Pond, the Gibbs free energy of nitrification was calculated for wide ranges of O_2_ concentrations (10^−4^ to 210 μM) and NH_4_^+^ concentrations (10^−5^ to 10 μM).

### DNA extraction.

Total genomic DNA present in basaltic rocks was extracted from ∼0.5 g of crushed sample using a FastDNA spin kit for soil (MP Biomedicals, Carlsbad, CA, USA) as described previously (19). DNA from the sediments was extracted from approximately 0.5 g of sample material using a PowerLyzer Max kit (Mobio) as previously described (25). DNA from the bottom seawater sample was extracted as described previously (22). To assess the potential of contamination introduced during the DNA extraction process, two extraction blanks (i.e., no sample material) were included in parallel. Furthermore, DNA from the plastic bag carrying the fluorescent microsphere deployed during drilling and DNA from the drill mud used during the operation were also extracted using the PowerLyzer Max kit (Mobio) as described elsewhere (19). DNA extracts from each rock and sediment sample were eluted into 100 μl PCR-grade double distilled water (ddH_2_O), while DNA extracts from the bottom seawater were eluted into 250 μl Tris-EDTA (TE) buffer. All DNA extracts were preserved at −20°C until further analysis.

### 16S rRNA gene amplicon preparation and sequence analysis.

16S rRNA gene amplicon libraries from the basalts and sediments were previously generated and sequenced and are reported elsewhere (19, 25). For this study, the 16S rRNA amplicon library from the bottom seawater was generated using an identical protocol (including primers, PCR conditions, and sequencing platform). In brief, 27 PCR cycles were used in the initial PCR amplification, while sample-specific tags and adaptors were attached during a second 7-cycle PCR. In both steps, the prokaryotic primers Uni519F (5′- CAGCMGCCGCGGTAA-3′) and 806R (5′- GACTACHVGGGTATCTAATCC-3′) were deployed to cover both the archaeal and bacterial domains. Resulting amplicons were sequenced on an IonTorrent Personal Genome Machine (PGM) in the Laboratory of Biodiversity, University of Bergen, Norway. The demultiplexed sequences were filtered and clustered into operational taxonomic units (OTUs) using USEARCH and UPARSE (74, 75). Quality filtering and trimming to 220 bp were performed with the ‘-fastq_filter’ command using options ‘-fastq_trunclen 220’ and ‘-fastq_maxee 1’. Chimeric sequences were detected and removed using the '-uchime_ref' command with the Gold database as the reference (available at https://drive5.com/uchime/gold.fa). *De novo* OTU clustering was performed at a cutoff of 97% nucleotide sequence similarity using the ‘-cluster_otus’ command. Taxonomic classification of OTUs was performed using the program CREST, applying the Lowest Common Ancestor algorithm and with SilvaMod as the reference database (76).

### Archaeal *amoA* gene screening, clone library construction, and sequence analysis.

The presence of archaeal (AOA) *amoA* genes was assessed by PCR using the primer pair CrenamoA23f/CrenamoA616r (77) under the following thermal conditions: 5 min initial activation step at 95°C, followed by 34 to 40 cycles consisting of 30 s denaturing at 95°C, 45 s annealing at 50°C, and 45 s extension at 72°C, with a final elongation step at 72°C for 10 min. Duplicate PCRs were performed for each sample in a 25-μl reaction volume consisting of 12.5 μl of 2× HotStart master mix (Qiagen, Germany), 1 μl of each forward and reverse primer (10 nM working solution), and 1 to 2 μl of DNA template. The PCR cycle numbers were 40 for all basaltic rocks and 35 for the seawater, and a minimum PCR cycle number (range, 34 to 40) was optimized for each sediment sample (Table S2). In addition to the AOA *amoA* gene, amplification of ammonia-oxidizing bacteria (AOB) *amoA* and nitrite oxidizing bacteria (NOB) *nxrB* genes was attempted on the DNA obtained from the basaltic rocks by the use of primer pairs amoA1F/amoA2R (78) and nxrB169f/nxrB638r (79), respectively, applying the thermal conditions described elsewhere (25).

10.1128/mSystems.00758-19.4 TABLE S2 PCR amplification strategies for the archaeal *amoA* gene in the North Pond habitats. Download Table S2, DOCX file, 0.01 MB. Copyright © 2020 Zhao et al. 2020 Zhao et al. This content is distributed under the terms of the Creative Commons Attribution 4.0 International license.

Cloning of AOA *amoA* genes was performed using the following procedure. Duplicate PCR products were pooled, purified using a GenElute PCR clean-up kit (Sigma-Aldridge, USA), and ligated into vectors using a Strata cloning kit (Agilent, USA) and transformed into Escherichia coli competent cells, according to the manufacturer’s instructions. Positive clones were subsequently selected using white-blue colony selection on LB-ampicillin agar plates. Forty positive clones were randomly picked from each sample and sequenced by Sanger sequencing on an ABI 3700 sequencer using BigDye chemistry reagents (Applied Biosystems, USA) in the Genewiz facilities in China (Genewiz, Beijing, China).

Recovered *amoA* gene sequences underwent primer deletion followed by chimera check and removal using the Functional Gene Pipeline and Repository analysis tool (FunGene; http://fungene.cme.msu.edu/). All quality-checked sequences were aligned using MAFFT-LINSi (80) and were imported into the mothur package (81). Operational taxonomic units (OTUs) were clustered based on a 5% nucleotide sequence divergence cutoff level (82), applying the furthest-neighbor-distance algorithm. Diversity indices, including the nonparametric richness estimators (*S*_obs_, *S*_chao1_, and Shannon) and Simpson diversity index, were calculated based on the OTU table. Coverage of the *amoA* gene libraries was calculated according to the equation *C* = [1 − (*n*/*N*)] × 100, where *n* is the number of OTUs generated based on only one sequence (representing singletons) and *N* is the number of clones examined.

### Quantitative PCR of archaeal *amoA* genes.

Quantification of archaeal *amoA* gene copy numbers in the basaltic rocks and bottom seawater was performed using the primer pair CrenamoA23f/CrenamoA616r (77) and thermal conditions as previously described for the overlying sediments (25). The archaeal fosmid 54d9 (83) was linearized by conventional PCR, and a 10× series of dilutions ranging from 1.5 to 1.5 × 10^5^ copies/μl were used as the standard. The efficiency level of the quantitative reaction for the standard series was 99%, with an *R*^2^ value of 0.94. All samples were analyzed in triplicate, and the final abundances of AOA *amoA* genes were normalized to copies per gram of sample material.

### Phylogenetic analyses of the two marker genes.

16S rRNA gene sequences affiliating with the Nitrosopumilaceae family were extracted from the larger microbial community data set. All OTU sequences were used as queries in BLASTn searches performed with the NCBI webserver to find their close relatives. To minimize the size of the phylogenetic tree, only the top two hits of length >1,200 bp were retained. All these sequences were aligned using MAFFT-LINSi (80) with AOA 16S rRNA genes compiled as described previously by Jørgensen et al. (84) supplemented with all the newly reported Thaumarchaeota genomes. Phylogenetic placement of the OTUs was evaluated by mapping our sequences onto the backbone maximum likelihood phylogenetic tree using the RAxML package (85). The nomenclature for the individual phylogenetic clades follows the descriptions provided in references 86 and 84. To explore whether the AOA phylotypes were unique to the subseafloor basalts at North Pond, we also included the 7 OTU sequences of Thaumarchaeota recovered from seafloor basalts at Dorado Outcrop (37) (see Fig. S2).

OTUs representing archaeal *amoA* genes (95% cutoff) were used in searches against the GenBank database using BLASTn (87) to find the most closely related sequences. All OTU sequences recovered from North Pond, the sequences corresponding to their closest environmental relatives, and the *amoA* gene sequences of known Thaumarchaeota genomes were included in the phylogenetic analysis. We also included the *amoA* gene sequences of two (NORP47 and NORP160) of the three Thaumarchaeota genomes (NORP164 has no 16S rRNA identified) recovered from North Pond crustal fluids. All sequences were aligned in MAFFT-LINSi (80) and were included to reconstruct a maximum likelihood phylogenetic tree using IQ-TREE v1.6.10 (88) with the GTR+F+I+G4 model (the best-fit model determined by ModelFinder [89]) and 1,000 ultrafast bootstraps. The individual *amoA* clades were designated following previously described nomenclature (30). To minimize confusion caused by the use of the different nomenclatures applied to the two trees, we matched the clades between the two trees based on the positions of known Thaumarchaeota genomes in the two trees.

### Data availability.

Raw reads of 16S rRNA genes generated in this study are deposited at the NCBI Sequence Read Archive under project number SRP070121 (basalt rocks) and project number PRJNA489438 (sediments and bottom seawater). Archaeal *amoA* gene sequences obtained in this study are deposited in GenBank under the following accession numbers: MF999267 to MF999866.
