# Supplementary material for: Indigenous Ammonia-Oxidizing Archaea in Oxic Subseafloor Oceanic Crust
Source: mSystems. 2020 Mar 10;5(2):e00758-19. doi: 10.1128/mSystems.00758-19 (PMC7065515; doi:10.1128/mSystems.00758-19)
Supplement: TABLE S1 [file mSystems.00758-19-st001.docx]

**Table S1. Proportion of ammonia-oxidizing bacteria (AOB) and nitrite-oxidizing bacteria (NOB) assessed by the 16S rRNA gene amplicon sequencing**

| **Sample ID** | **% Total AOB** | **AOB reads #** | | | **% Total NOB** | **NOB reads #** | |
| --- | --- | --- | --- | --- | --- | --- | --- |
|  |  | ***Nitrosospira*** | ***Nitrosomonas*** | ***Nitrosococcus*** |  | ***Nitrospira*** | ***Nitrospina*** |
| **Bottom seawater** | | | | | | | |
| BW_2014 | 0 | 0 | 0 | 0 | 0.05 | 0 | 21 |
| **U1383E sediments** | | | | | | | |
| 83E_1H-1 | 2.36 | 104 | -- | 639 | 3.17 | 909 | 88 |
| 83E_2H-1 | 0.05 | 6 | -- | 7 | 3.01 | 47 | 670 |
| 83E_2H-2 | 0.44 | 15 | -- | 77 | 2.68 | 270 | 288 |
| 83E_2H-4 | 0.12 | 5 | -- | 29 | 1.18 | 3 | 324 |
| 83E_2H-6 | -- | -- | -- | -- | -- | -- | -- |
| 83E_3H-3 | 0.19 | -- | 4 | -- | 3.52 | 1 | 73 |
| 83E_3H-6 | 0.70 | 4 | 1 | 1 | 4.09 | 5 | 30 |
| 83E_4H-2 | 0.53 | 53 | -- | 118 | 2.74 | 813 | 78 |
| 83E_4H-3 | 0.34 | 10 | -- | 110 | 1.19 | 212 | 211 |
| 83E_4H-4 | -- | -- | -- | -- | -- | -- | 1 |
| 83E_4H-5 | -- | -- | -- | 2 | 0.05 | -- | 16 |
| 83E_4H-6 | -- | -- | -- | -- | 2.50 | -- | 542 |
| 83E_4H-7 | 0.13 | 34 | -- | -- | 0.16 | 2 | 12 |
| 83E_5H-2 | -- | -- | -- | -- | 2.22 | 2 | 5380 |
| 83E_5H-4 | -- | -- | -- | -- | 0.92 | -- | 267 |
| 83E_5H-6 | -- | -- | -- | -- | 1.84 | -- | 661 |
| 83E_6H-2 | -- | -- | -- | -- | 1.03 | -- | 246 |
| 83E_6H-5 | -- | -- | -- | -- | 0.40 | 16 | 139 |
| 83E_6H-6 | -- | -- | -- | -- | 0.45 | 1 | 117 |
| **U1383C_basalts** | | | | | | | |
| **83C_2R_2E** | 0.05 | 9 | -- | -- | -- | -- | -- |
| 83C_3R_1B | -- | -- | -- | -- | -- | -- | -- |
| **83C_4R_1B** | -- | -- | -- | -- | -- | -- | -- |
| **83C_5R_1B_I** | 0.19 | 29 | -- | -- | 0.07 | 11 | -- |
| 83C_5R_1B_II | -- | -- | -- | -- | 0.04 | 8 | -- |
| 83C_6R_1A | 0.07 | 16 | -- | -- | 0.02 | -- | 4 |
| 83C_10R_1A | 0.13 | 34 | -- | -- | 0.72 | -- | 186 |
| 83C_10R_1D | -- | -- | -- | -- | 0.06 | -- | 10 |
| 83C_11R_1C | -- | -- | -- | -- | -- | -- | 1 |
| **83C_19R_1B** | -- | -- | -- | -- | -- | -- | -- |
| **83C_19R_1A** | -- | -- | -- | -- | -- | -- | -- |
| 83C_20R_1A | -- | -- | -- | -- | 0.01 | -- | 1 |
| 83C_24R_1B | -- | -- | -- | -- | -- | -- | -- |
| 83C_24R_1A | -- | -- | -- | -- | -- | -- | -- |
| 83C_27R_1A | -- | -- | -- | -- | 0.03 | -- | 3 |
| 83C_29R_1A | -- | -- | -- | -- | -- | -- | -- |
| **83C_30R_1A** | -- | -- | -- | -- | -- | 1 | -- |
| **U1382A_basalts** | | | | | | | |
| **82A_2R_1C** | 0.07 | 6 | -- | -- | 0.09 | 8 | -- |
| 82A_3R_2B | -- | -- | -- | -- | 0.02 | -- | 3 |
| 82A_3R_3A | -- | -- | -- | -- | 0.01 | -- | 2 |
| 82A_3R_4B | -- | -- | -- | -- | -- | -- | -- |
| 82A_4R_1B | -- | -- | -- | -- | 0.08 | 10 | 2 |
| **82A_5R_1B** | 0.24 | 47 | -- | -- | -- | -- | -- |
| 82A_6R_1A | -- | -- | -- | -- | 0.47 | 97 | -- |
| 82A_7R_2B | -- | -- | -- | -- | 0.01 | -- | 1 |
| **82A_8R_1B** | -- | -- | -- | -- | -- | -- | -- |
| 82A_8R_1A | -- | -- | -- | -- | -- | -- | -- |
| 82A_8R_2F | -- | -- | -- | -- | -- | -- | -- |
| 82A_8R_3G | -- | -- | -- | -- | 0.01 | 1 | -- |
| 82A_8R_4D | -- | -- | -- | -- | -- | -- | -- |
| **82A_9R_1C** | -- | -- | -- | -- | 0.19 | 32 | -- |
| 82A_10R_3D | 0.23 | 42 | -- | -- | -- | -- | -- |
| 82A_12R_1A | -- | -- | -- | -- | -- | -- | -- |

-- Not detected

Samples_highlighted_in red were used in archaeal *amoA* gene_clone_library_construction.
