# Supplementary figures and images for: Indigenous Ammonia-Oxidizing Archaea in Oxic Subseafloor Oceanic Crust
Source: mSystems. 2020 Mar 10;5(2):e00758-19. doi: 10.1128/mSystems.00758-19 (PMC7065515; doi:10.1128/mSystems.00758-19)

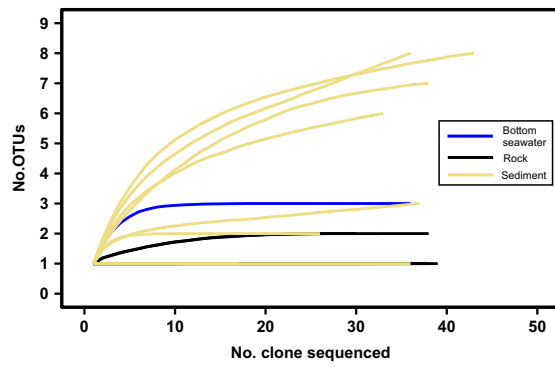

Supplement: FIG S1 [file mSystems.00758-19-sf001.pdf]

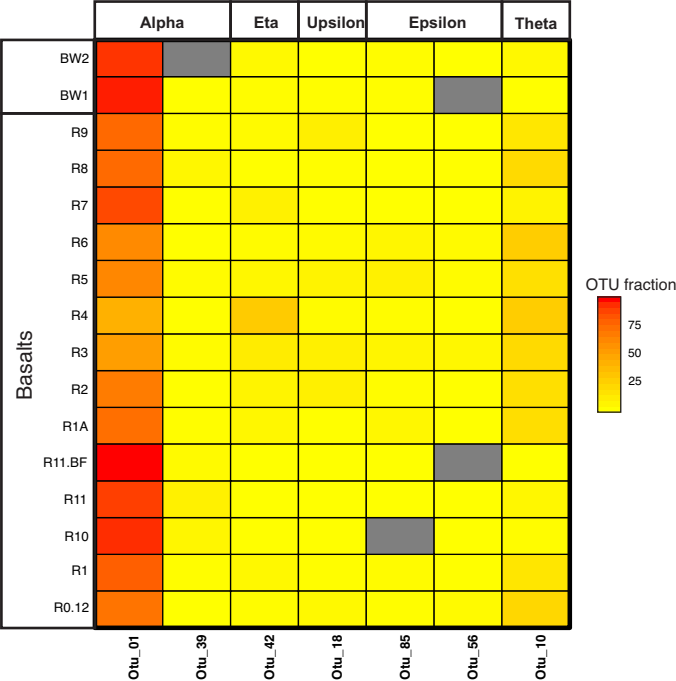

Supplement: FIG S2 [file mSystems.00758-19-sf002.pdf]
