# Supplementary material for: Indigenous Ammonia-Oxidizing Archaea in Oxic Subseafloor Oceanic Crust
Source: mSystems. 2020 Mar 10;5(2):e00758-19. doi: 10.1128/mSystems.00758-19 (PMC7065515; doi:10.1128/mSystems.00758-19)
Supplement: TABLE S2 [file mSystems.00758-19-st002.docx]

**Table S2. PCR amplification strategies of the archaeal *amoA* gene in the North Pond habitats**

| Niches | Horizon | Depth/  mbsf | Amplification | |
| --- | --- | --- | --- | --- |
|  |  |  | PCR_cycle_# | Template  volume/µl |
| Bottom_seawater | -- | 0 | 35 | 1 |
| Surficial sediments (U1383E) | U1383E_1H-1 | 0.1 | 34 | 1 |
|  | U1383E_2H-1 | 3.1 | 36 | 1 |
|  | U1383E_2H-2 | 4.6 | 36 | 1 |
|  | U1383E_4H-2 | 22.0 | 37 | 1 |
| Basal sediments | U1383E_4H-7 | 29.5 | 36 | 1 |
|  | U1383E_6H-2 | 41.4 | 40 | 2 |
|  | U1383E_6H-5 | 45.7 | 40 | 2 |
|  | U1383E_6H-6 | 47.0 | 40 | 2 |
| Basalt in U1383C | U1383C_2R_2E | 72.2 | 40 | 2 |
|  | U1383C_4R_1B | 87.6 | 40 | 2 |
|  | U1383C_5R_1B_I | 97.0 | 40 | 2 |
|  | U1383C_19R_1B | 212.2 | 40 | 2 |
|  | U1383C_19R_1A | 212.0 | 40 | 2 |
|  | U1383C_30R_1A | 304.0 | 40 | 2 |
| Basalt in U1382A | U1382A_2R_1 | 110.6 | 40 | 2 |
|  | U1382A_5R_1B | 133.2 | 40 | 2 |
|  | U1382A_8R_1B | 142.4 | 40 | 2 |
|  | U1382A_9R_1C | 171.2 | 40 | 2 |
